# Optimizing treatment success in multiple sclerosis

**DOI:** 10.1007/s00415-015-7986-y

**Published:** 2015-12-24

**Authors:** Tjalf Ziemssen, Tobias Derfuss, Nicola de Stefano, Gavin Giovannoni, Filipe Palavra, Davorka Tomic, Tim Vollmer, Sven Schippling

**Affiliations:** MS Center Dresden, Center of Clinical Neuroscience, Neurological Clinic, University Hospital Carl Gustav Carus, Dresden University of Technology, Fetscherstrasse 74, 01307 Dresden, Germany; Department of Medicine, Surgery and Neuroscience, University of Siena, Siena, Italy; Queen Mary University London, Barts and The London School of Medicine and Dentistry, London, UK; Neurology-Neuroimmunology Department, Multiple Sclerosis Centre of Catalonia (Cemcat), Vall d’Hebron University Hospital, Barcelona, Spain; Novartis Pharma AG, Basel, Switzerland; University of Colorado Health Sciences Center, Aurora, CO USA; Department of Neurology, Neuroimmunology and Multiple Sclerosis Research, University Hospital Zurich, University of Zurich, Zurich, Switzerland

**Keywords:** Brain atrophy, Disability evaluation, Drug therapy, Multiple sclerosis

## Abstract

Despite important advances in the treatment of multiple sclerosis (MS) over recent years, the introduction of several disease-modifying therapies (DMTs), the burden of progressive disability and premature mortality associated with the condition remains substantial. This burden, together with the high healthcare and societal costs associated with MS, creates a compelling case for early treatment optimization with highly efficacious therapies. Often, patients receive several first-line therapies, while more recent and in part more effective treatments are still being introduced only after these have failed. However, with the availability of highly efficacious therapies, a novel treatment strategy has emerged, where the aim is to achieve no evidence of disease activity (NEDA). Achieving NEDA necessitates regular monitoring of relapses, disability and functionality. However, there is only a poor correlation between conventional magnetic resonance imaging measures like T2 hyperintense lesion burden and the level of clinical disability. Hence, MRI-based measures of brain atrophy have emerged in recent years potentially reflecting the magnitude of MS-related neuroaxonal damage. Currently available DMTs differ markedly in their effects on brain atrophy: some, such as fingolimod, have been shown to significantly slow brain volume loss, compared to placebo, whereas others have shown either no, inconsistent, or delayed effects. In addition to regular monitoring, treatment optimization also requires early intervention with efficacious therapies, because accumulating evidence shows that effective intervention during a limited period early in the course of MS is critical for maintaining neurological function and preventing subsequent disability. Together, the advent of new MS therapies and evolving management strategies offer exciting new opportunities to optimize treatment outcomes.

## Introduction

Although recent years have seen great advances in the treatment of multiple sclerosis (MS), with an increasing number of disease-modifying therapies (DMTs) becoming available, it remains a potentially serious and debilitating condition as none of the current treatments halts or cures the disease. A broad range of neurological functions may be affected, including vision, gait and motor function, cognition, coordination, and balance, as well as bladder, bowel and sexual function [1]. Cognitive impairment, for example, is present in up to 82 % of patients with MS [2–5]: it can be detected in the earliest stages of the disease [5], and adversely affects employment, activities of daily living, and social function [3, 6, 7]. Furthermore, in most cases, MS causes progressive disability, which can involve both motor and cognitive function and has a detrimental impact on patients’ quality of life [8]. Indeed, there is evidence that the impact of MS-related fatigue, unemployment and limited mobility on quality of life is greater than that associated with other causes of disability [9, 10]. MS-related disability is a major driver of the substantial healthcare and social costs associated with the condition [7]: European [11] and US [9, 12] data suggest that approximately 40–44 % of total MS-related costs result from lost productivity.

**Fig. 1:**
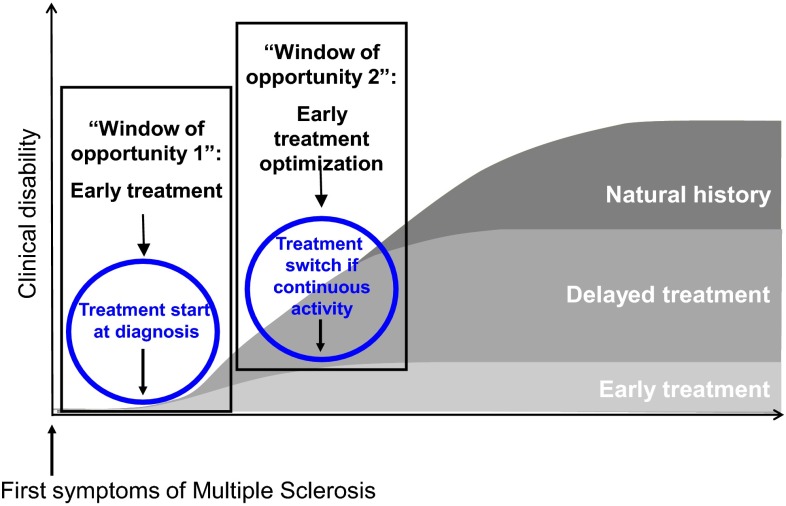
The ‘windows of opportunity’ for treatment optimization in MS. Early initiation of treatment, and prompt intervention if disease activity persists despite initial treatment, are both critical to optimizing treatment outcomes. In both cases, there is only a limited period during which intervention will be effective. Adapted with permission from Tintoré [22]

In addition to the physical and cognitive impairment associated with MS, life expectancy in people with MS is on average 8–12 years shorter than in the general population [13–16]. Up to approximately 78 % of people with MS die of disease-related complications such as respiratory tract infections or accidents [16–19]. This burden of disability and premature mortality, and the substantial economic costs associated with the condition, create a compelling case for early intervention and early treatment optimization with the more efficacious treatments that are now becoming available. At present, it is common practice in many countries for patients to receive several first-line therapies, such as interferon (IFN)-β, glatiramer acetate, teriflunomide or dimethyl fumarate (DMF), before therapies with greater efficacy, such as fingolimod, natalizumab or alemtuzumab, are tried following failure of these first-line agents [20]. However, there is increasing evidence that both early intervention after diagnosis, and early treatment optimization in the event of insufficient response to initial treatment (Fig. 1), are critical to achieving a favourable outcome and reducing the progressive burden imposed by MS on the patients, their families, and society as a whole [21].

The question of how best to intervene early in MS in order to achieve an optimal outcome was discussed at a round-table meeting in Barcelona, Spain, in June 2013. The key outcomes from this meeting are summarized in this paper.

## Monitoring disease activity in multiple sclerosis

**Fig. 2:**
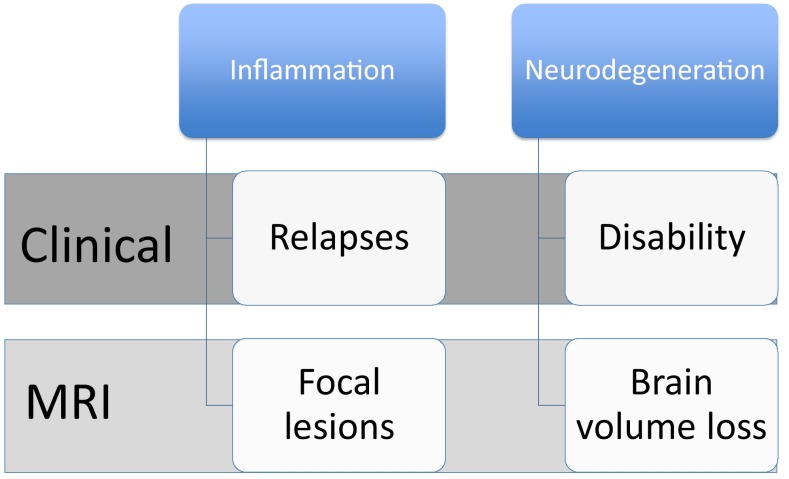
Associations between inflammatory and degenerative processes in MS and the clinical and MRI features of the disease

**Fig. 3:**
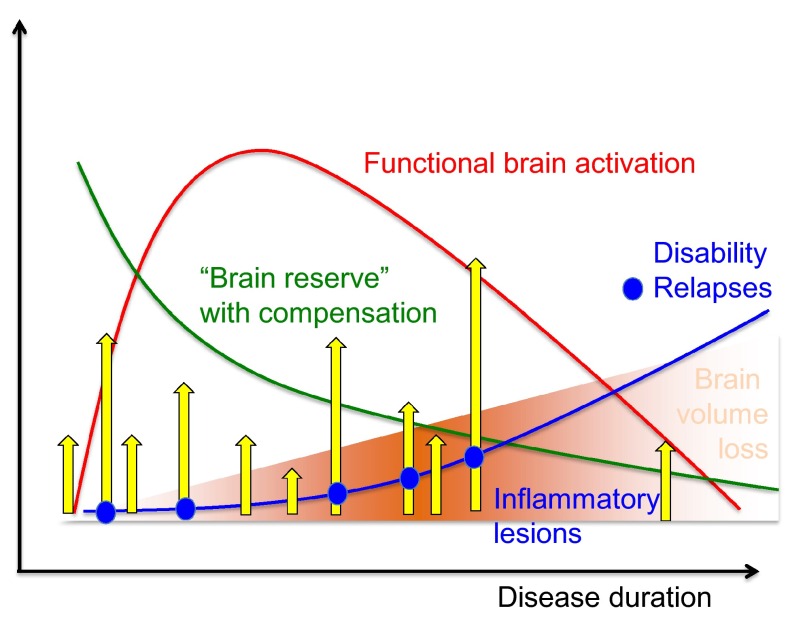
The relationship between relapses, inflammation and disability in MS. The disease process in MS is characterized by both inflammation and progressive neuroaxonal damage. Importantly, such damage may be present in the early stages of MS, but may be masked by compensatory mechanisms; hence, progressive damage may go unrecognized until it is too late for intervention to be beneficial. As the disease progresses, the balance between degenerative and reparative processes shifts, resulting in progressive neuroaxonal degeneration and increasing disability

**Fig. 4:**
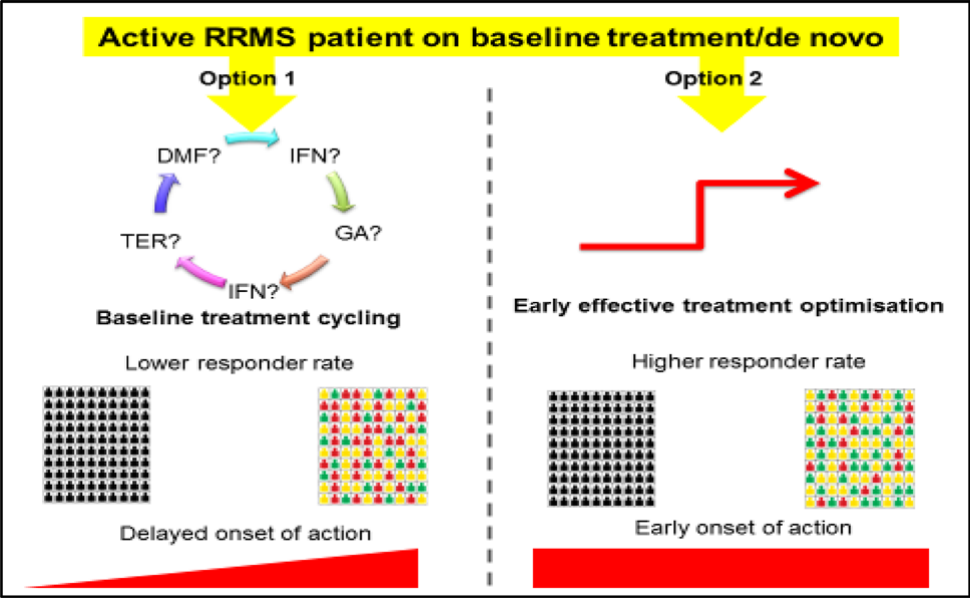
Potential treatment strategies in MS. In patients with little evidence of disease activity at baseline, treatment can be started with conventional first-line therapies such as IFN-β, glatiramer acetate, DMF or teriflunomide. Treatment should be monitored every 6–12-months. For patients with highly active disease at baseline or rapidly evolving severe disease (≥2 disabling relapses in 1 year, with at least one Gd+ lesion on T1-weighted MRI or a significant increase in lesion load on T2-weighted MRI), newer agents can be used as first-line therapy. The main differences between these two strategies are the higher responder rate and the earlier onset of action with the latter, which has to be evaluated for each individual patient

Monitoring MS disease activity is key to achieving optimal outcomes. However, the heterogeneity of the disease, and the complexity of the underlying biological mechanisms, can render this challenging. MS pathology is characterized by two major hallmarks: inflammation and progressive neuroaxonal damage [23–27]. From a clinical perspective, inflammation is infrequently associated with the subacute onset of clinical signs and symptoms and focal lesions on magnetic resonance imaging (MRI) that usually show temporary permeability of the blood–brain barrier, reflected by contrast enhancement at sites of acute inflammation. By contrast, axonal degeneration and loss of neurons are associated with sustained disability and evidence of brain or spinal cord atrophy on MRI over time (Fig. 2) [28]. Axonal transection is a consistent pathological feature of acute MS lesions, and the incidence of neuronal damage correlates with the extent of inflammation within the lesion [25]. Importantly, such damage may be present in the early stages of MS [27]. It can, however, be masked by mechanisms such as recruitment of other neuronal pathways or cortical remodelling, that compensate for functional loss; hence, progressive damage may go unrecognized until it is too late for an intervention to be beneficial [29, 30]. As the disease progresses, the balance between degenerative and reparative processes shifts, resulting in progressive neuroaxonal degeneration and increasing disability (Fig. 3). Hence, clinical disease monitoring in MS should have three elements: disease activity as manifested in relapses (reflecting inflammation), disability (reflecting neuroaxonal loss) and functionality (reflecting the degree of compensation or cerebral reserve) (Fig. 4).

### Monitoring MS by clinical parameters: relapses versus disability

In the majority of MS patients, the disease initially takes a relapsing–remitting course (RRMS), characterized by acute symptomatic relapses followed by periods of variable recovery. In the absence of treatment, more than 50 % of patients with RRMS will develop progressive disability after approximately 15 years [31].

Natural history studies have provided important insights into the determinants of disability progression in early MS. One such study showed a significant association between relapses occurring in the early stages of MS and long-term disability, which was primarily driven by an increasing risk of SPMS and, to a lesser extent, by an effect of frequent relapses on the rate of progression [32]. A further study [33] found that age at onset of MS, residual deficits after a first relapse, and the number of relapses during the first 2 years were predictive of the time to a Disability Status Score (DSS) of 3 (moderate disability), but not of the time from DSS 3 to DSS 6 (requiring assistance to walk). The authors suggested that these findings would be consistent with a two-phase process of progressive disability, in which the first stage is related to focal inflammation that is amenable to treatment, whereas the second stage is independent of current inflammation and may be related to diffuse neurodegeneration [33].

### Monitoring MS by MRI: inflammatory activity versus destructive markers

Conventional MRI techniques, such as T2-weighted imaging and gadolinium (Gd)-enhanced T1-weighted imaging (Table 1) offer good sensitivity in assessing the location and temporal evolution of demyelinating plaques in the brain and spinal cord of MS patients; indeed, these techniques are considered to represent the ‘gold standard’ for diagnosing MS and monitoring the response to treatment [31, 34]. However, due to the limited pathological specificity of these techniques, they provide little information about the underlying inflammatory process in MS, and show only weak correlations with clinical measures of disability [31, 34].

**Table 1:**
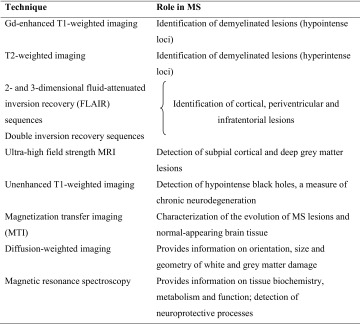
Conventional and emerging techniques used in the assessment of MS [34]

Alternatively, this apparent lack of correlation between conventional MRI measures and clinical disability could also be due to the low sensitivity of clinical measures of disability applied in routine clinical practice. For example, the widely used Expanded Disability Status Scale (EDSS) reflects the level of damage that has already occurred, and provides no information about the underlying neurodegenerative and reparative processes. Other MRI techniques, such as magnetization transfer (MTR), diffusion-tensor imaging (DTI) and proton magnetic resonance spectroscopy (MRS) (Table 1), appear to provide better and more quantitative measures of higher pathological specificity for hallmarks of the disease such as demyelination and remyelination (MTR, DTI) or axonal degeneration (MRS, DTI). Moreover, they show better correlations with standard measures of clinical disability [31, 34].

In addition to focal MRI lesions, MRI measurements of more diffuse brain atrophy have emerged in recent years as a promising measure of MS-related neuroaxonal damage, that could in principle be used to measure treatment effects [35–37]. Brain atrophy is a characteristic feature of MS, occurring in the earliest stages and progressing throughout the course of the disease [38]. In people with MS, brain atrophy progresses at a rate of approximately 0.5–1.0 % per year, compared with 0.2–0.4 % in healthy individuals [35, 39], although it should be noted that it is unclear whether atrophy progresses in a linear fashion in individual patients. Of note, changes in brain volume in MS can reflect diverse pathophysiological mechanisms, including changes in inflammatory oedema, neuronal or axonal loss, de- and remyelination, and changes in glial cell number and volume [35, 39].

Several studies have shown that, at a group level, brain atrophy in MS patients appears to be predictive of subsequent disability [40, 41]. Indeed, it has been suggested that measurement of brain atrophy may be the best predictor of subsequent disability in MS patients [37, 42, 43]. Although atrophy affects both grey and white matter regions of the brain [37, 42], there is evidence that grey matter (cortical and deep grey matter) atrophy is more closely related to long-term disability than white matter atrophy. In one study, for example, grey matter atrophy showed significant correlations with disability measured either by the EDSS or the MS functional composite (MSFC), whereas no such correlations were seen with white matter atrophy; furthermore, changes in the grey matter fraction accounted for a greater proportion of the variability in clinical findings than changes in white matter [44]. Importantly, a recent pathological study has shown that plaque-like primary demyelinating cortical lesions are specific to MS, and are not seen in other neuroinflammatory disorders such as tuberculous meningitis or chronic purulent meningitis [45].

Recent studies have shown that the combination of brain atrophy measures and MRI lesion load is a strong predictor of long-term disability. In a study of 261 MS patients in whom EDSS assessments were available at baseline and after 10 years’ follow-up, and in whom MRI investigations were performed over 1–2 years after baseline, the combination of these MRI measures was strongly predictive of EDSS scores after 10 years (*R*^2^ = 0.74); furthermore, central atrophy was predictive of long-term disability in patients with minimal impairment (EDSS 0–3.5) at baseline, whereas T2 lesion volume was predictive in patients with moderate impairment (EDSS 4–6) at baseline [46].

A recent meta-analysis, including data from more than 13,500 patients enrolled in 13 randomized controlled trials, has investigated the relationship between changes in brain atrophy and disease progression during treatment of RRMS [47]. Treatment effects on both brain atrophy and active MRI lesions (defined as new or enlarging T2 lesions) were significantly and independently correlated with effects on disability progression at group level, and the correlation was strongest when both MRI endpoints were included in a multivariate model.

The available DMTs appear to differ in their effects on brain atrophy (Table 2) [36, 50–70], although it should be noted that direct comparisons are difficult between trials because of the heterogeneity of patient populations and methods applied to measure brain volume used in different studies. During treatment with many DMTs, an apparent decrease in brain volume (pseudoatrophy) occurs during the first 6–9 months, but a significant decrease in atrophy rate, compared with placebo, occurs during the second year of treatment with some agents [56, 61, 63, 71–77].

**Table 2:** Immediate and delayed treatment effects on brain volume changes in the double-blind phases of trials of disease-modifying therapies in RRMS [48–70]

Drug	Numbers of patients	Global effect on brain volume	Immediate effect on brain volume	Delayed effect on brain volume
*Placebo*-*controlled studies*				
Interferon β-1a [48–51]	172, 382	No	No	Yes
Glatiramer acetate [52–55]	27 (subcohort), 207, 980	No	No^a^	NA^b^
Fingolimod [56–58]	1033, 1153	Yes	Yes	Yes
Dimethyl fumarate [59, 60]	540, 681	Yes^c^	No^d^	Yes^d^
Teriflunamide [63]	1074	No	No	No
Laquinimod [64, 65]	1106, 1331	Yes	NA	NA
Natalizumab [61, 62]	942, 1003	No	No	Yes
*Active comparator studies*				
Interferon versus glatiramer acetate [66–68]	460, 1008, 2096	Yes (GA)^e^	Yes (GA)^e^	Yes (GA)^e^
Fingolimod versus im IFN β-1a [57]	1153	Yes (FTY)	Yes (FTY)	NA^f^
Alemtuzumab versus sc IFN β-1a [69, 70]	334, 581, 840	Yes (AL)	NA^g^	NA^g^

Adapted from Vidal-Jordana et al. [48]

*AL* alemtuzumab, *IFN* interferon, *im* intramuscular, *GA* glatiramer acetate, *NA* not applicable, *sc* subcutaneous

^a^Baseline to 9 months

^b^Open-label data: a significant effect of glatiramer acetate was observed in months 9–18 in the early treatment arm

^c^Only for twice-daily dosing in the DEFINE trial; brain volume was assessed during the 6–24 month period

^d^Only for twice-daily dosing in the CONFIRM trial: no data available for the DEFINE trial

^e^Data only from the REGARD trial, no *P* values reported; no significant differences were observed in the BEYOND AND COMBIRx trials

^f^No data available beyond 12 months

^g^The two CARE-MS trials only assessed brain volume changes from baseline to 24 months

At present, brain atrophy is not measured routinely in MS centres and is not used to monitor treatment. Hence, the use of brain atrophy as an outcome measure in MS will require standardization of MRI acquisition and post-processing procedures to allow comparisons of scans obtained at different times during the course of MS and at different centres. In clinical trials, brain volume should be measured at 3–6 month intervals to identify pseudoatrophy [78, 79], while in routine clinical practice scans should be taken 6 months after starting DMTs to establish a baseline for assessments of brain atrophy that is less likely confounded by pseudoatrophy effects. SIENA (Structural Image Evaluation, using Normalization, of Atrophy), usually used to measure brain volume loss in clinical trials, could potentially be incorporated into MS management but would still necessitate a technology and staff infrastructure not necessarily available in MS centers. However, other simpler techniques such as measurement of third ventricular width, lateral ventricle volume and corpus callosum index might provide alternative options once validated in clinical routine [37, 42, 80, 81].

### Monitoring MS by patient-reported outcomes

From a patient’s perspective, a treatment may be considered to be a failure if it produces adverse events that affect everyday quality of life. This would suggest that worsening in patient-reported outcomes related to fatigue, depression, cognitive dysfunction, mobility, sexual function or bowel/bladder function should also be included in definitions of treatment failure [1]. Similarly, in view of the significant impact of MS-related disability on quality of life [8], changes in quality of life should be considered an important outcome in MS treatment (Fig. 4). 

It may be anticipated that patient-reported outcomes will become increasingly important in MS management as the focus of treatment moves to the prevention or delay of disability, rather than clinical relapses or MRI measures of disease activity [82]. It will therefore be necessary to validate such outcome measures in clinical trials and routine practice [83]. A recent study has found that two widely used outcome measures, the MS Impact Scale (MSIS-29) and the Hamburg Quality of Life Questionnaire in MS (HAQUAMS), are able to differentiate between MS patients with different degrees of functional impairment, with moderate correlations between these instruments and conventional disability measures such as the EDSS and the MSFC [84].

### Combining monitoring strategies in MS treatment: the NEDA concept

As noted above, current practice in MS is to start with first-line therapies and then introduce more efficacious agents if the response is inadequate or if first-line therapy is poorly tolerated [19]. This approach is enshrined in current MS management guidelines from a number of European countries [85, 86]. In recent years, however, a new strategy has emerged, ‘treating to target,’ where the aim is to achieve no evidence of disease activity (NEDA). This may be defined as absence of relapses, disability progression and MRI measures of disease activity including new Gadolinium enhancing and new or newly enlarging T2 lesions [87]. There is evidence that MS patients treated to target of NEDA have better outcomes than those with clinical or subclinical breakthrough disease, and hence it has been recommended by some that this approach should be incorporated into routine clinical practice [88]. A recent long-term (up to 7 years) study found that NEDA status at 2 years had optimal prognostic value, although NEDA was difficult to sustain over the longer term, even with treatment [89]. In this study, NEDA was defined as a composite of absence of relapses, no EDSS progression and no new or enlarging T2 or T1 Gd-enhancing lesions on annual MRI. However, it has been argued that such a focus on clinical and MRI measures does not adequately reflect patients’ needs in routine clinical practice [90].

In view of such considerations, it is anticipated that the definition of NEDA is likely to evolve as evidence accumulates to support the incorporation of additional outcome measures [88]. For example, there is increasing evidence that the absence of brain atrophy, as measured by MRI, may also be a valid criterion for NEDA. This view is based on the evidence, discussed above, that measures of brain atrophy, despite methodological limitations, appear to be a clinically useful marker of neuroaxonal damage in MS; indeed, early brain atrophy has recently been shown to be predictive of response to IFN-β treatment [91]. The combination of relapses, disability progression and conventional MRI measures with assessment of brain volume loss has been termed NEDA-4 [92]. In an analysis of two pivotal trials with fingolimod, the addition of brain volume loss increased the stringency of the NEDA measure without affecting the sensitivity of the measurement to treatment effects [92]. However, regular MRI monitoring of brain volume may not be currently feasible in routine clinical practice due to limited availability of the technological infrastructure and trained staff as indicated above.

The increasing focus on NEDA as an aim of MS therapy implies that regular, systematic, monitoring should be a central aspect of the management of the condition, and this is reflected in recent Canadian guidelines that recommend the implementation of MRI monitoring, ultimately advocating implementation of NEDA-4 as an aspirational goal [93]. These guidelines recommend regular MRI follow-up, beginning at 3–6 months after initiation of treatment, at 6–12 months after the reference scan and annually thereafter [93].

## The importance of early diagnosis and early treatment in MS

Evidence is accumulating to support the assumption that there is a period early in the course of MS during which treatment is most efficacious, and that effective treatment during this period appears to be critical for maintaining long-term neurological function and preventing subsequent disability and premature mortality over the lifetime of the patient.

Several clinical trials have provided proof of concept for an early window of first treatment intervention in clinically isolated syndrome (CIS). Results from the 2-year blinded phase of the BENEFIT study [94] in patients with a first event suggestive of MS showed that the time to confirmed progression on the EDSS was significantly longer in those receiving early treatment than in those who were on placebo, and so had a delayed start of treatment, and the risk of progression was reduced by 40 % [hazard ratio (HR) 0.60, 95 % confidence interval (CI) 0.39–0.92, *P* = 0.022]. The authors noted that, although the delay in treatment was equivalent to just a single clinical event, this was nevertheless sufficient to influence the subsequent accumulation of disability, and that the delay in progression associated with early treatment could be considered to be clinically relevant [94]. A subsequent analysis after 5 years of follow-up showed that the rate of progression to clinically definite MS was significantly lower with early treatment than with delayed treatment, although the risk of confirmed progression of disability did not differ between the groups and mean EDSS scores were low [73]. Other IFN-β studies [95–99] showed that, compared with placebo, early intervention significantly reduced the risk of progression to clinically definite MS in patients with a first clinical event suggestive of MS, and similar results have been obtained with glatiramer acetate [100] and teriflunomide [101].

Further evidence for a critical period for early intervention in MS comes from a study with alemtuzumab, which involved both an SPMS and an RRMS cohort [102]. The mean (±SD) disease duration at the start of alemtuzumab treatment was 11.2 ± 6.1 years in the secondary progression cohort, of which an average of 3.6 ± 2.6 years had been spent in the progressive phase, whereas in the RRMS group the mean duration of disease prior to treatment was 2.7 ± 2.9 years. In the RRMS cohort, treatment with alemtuzumab significantly reduced relapse rates, prevented the accumulation of disability, and allowed some patients to recover function as measured by the EDSS; by contrast, in patients with SPMS alemtuzumab suppressed inflammation and slowed (but did not prevent) progressive disability, and there was little recovery of function. These findings were attributed by the authors to the beneficial effects of early rescue of neurons from an inflammatory environment [102]. Additional evidence comes from the results of an extension phase to the FREEDOMS study, in which patients who received placebo during the double-blind phase were switched to fingolimod. Although these patients showed significant clinical improvements, including reductions in relapse rates, disability progression and brain atrophy, following initiation of fingolimod, these benefits were less marked than in patients who received fingolimod treatment from the start of the study [103].

## Early treatment optimization

### MRI lesions and clinical endpoints

A recent 15-year follow-up study of RRMS patients who received IFN-β-1a during a pivotal clinical trial has shown that the presence of at least two Gd-enhancing lesions over the 2-years of treatment in the IFN arm of the study was strongly predictive of EDSS worsening [104]. In a further study, the presence of two or three measures of disease activity (new MRI lesions, relapses or confirmed 1-point EDSS progression) during the first year of IFN-β treatment was predictive of a subsequent poor response to therapy [105].

A scoring system for MS disease activity was described by Río et al. [106], who analysed data from 222 patients with RRMS who had received IFN-β1a for at least 1 year. This system was based on measurements of clinical relapses, disability progression (increase of 1 EDSS point confirmed at 6 months) and active MRI lesions (≥2 new T2 or Gd-enhancing lesions) 1 year after the start of treatment. Patients who met at least two of these criteria were more likely to experience progressive disability or relapses during the subsequent 2 years than those who did not. However, relapses or MRI criteria alone were not predictive of new disease activity or disease progression. By contrast, Prosperini et al. [107] found that the 4-year outcomes of patients with isolated MRI activity after the first year of IFN-β therapy did not differ from those fulfilling the European Medicines Agency (EMA) criteria for second-line treatment escalation. This would suggest that MRI alone might be a good predictor of outcome.

A modified version of the Río scoring system has recently been published, based on relapses and focal MRI activity only [108, 109]. Validation of this system in the dataset used to develop the original Río system resulted in a 24 % probability of disease progression in patients considered to be at low risk of progression, a 33 % probability in medium-risk patients, and a 65 % probability in high-risk patients; a subsequent study showed that more efficient classification of medium-risk patients could be achieved by further MRI and clinical evaluation 6 months after the first year of therapy [110].

In a long-term (16 years) retrospective follow-up of a pivotal IFN-β trial, baseline EDSS scores correlated with both physical and cognitive outcome (*R*^2^ = 0.22 and 0.12, respectively, *P* < 0.0001 for both), while accumulation of disability during the course of the study correlated significantly with physical outcome (*R*^2^ = 0.11, *P* < 0.0001), but not with cognition [111]. By contrast, baseline MRI measures of atrophy and lesion burden correlated with cognitive outcome (*R*^2^ = 0.21, *P* < 0.0001), but not with physical outcome. These findings offer support for the hypothesis that long-term outcome in MS is influenced at least in part by disease activity during the initial years of the disease.

The growing evidence, discussed above, that there is only a limited window of opportunity for effective intervention in MS with currently available drugs would suggest that regular monitoring during treatment with DMTs, and prompt intervention in cases of suboptimal response or treatment failure, are essential to prevent long-term disability. (Although it should be noted that the impact of early treatment switching has, to date, been studied only in patients receiving IFN-β.) As described previously, at present it is common for a patient to receive several first-line therapies, with escalating doses or treatment switches if the responses are inadequate, before more efficacious therapies are tried [20]. The available evidence suggests that switching to a different class of DMT (either as another first-line therapy or as second-line treatment) is more effective than dose escalation or switching to another member of the same class [83, 84, 112–117]. For example, in the CARE-MS II study, treatment with alemtuzumab reduced relapse rates and disability in RRMS patients who had previously experienced at least one relapse during first-line treatment with IFN β-1a or glatiramer acetate [117]. However, there is also evidence that initiating treatment with newer agents may be more effective than introducing these agents as second-line treatment. For example, in a randomized extension to the TRANSFORMS study, patients who received fingolimod from the start of the trial showed better clinical and MRI outcomes than those originally randomized to IFN-β-1a and subsequently switched to fingolimod during the extension phase [118]. Currently, highly effective DMTs such as fingolimod, natalizumab and alemtuzumab are mainly licensed for the first-line treatment of patients with highly active MS; further clinical data, including cost-effectiveness data, will be needed to support the early use of such therapies [119].

**Fig. 5:**
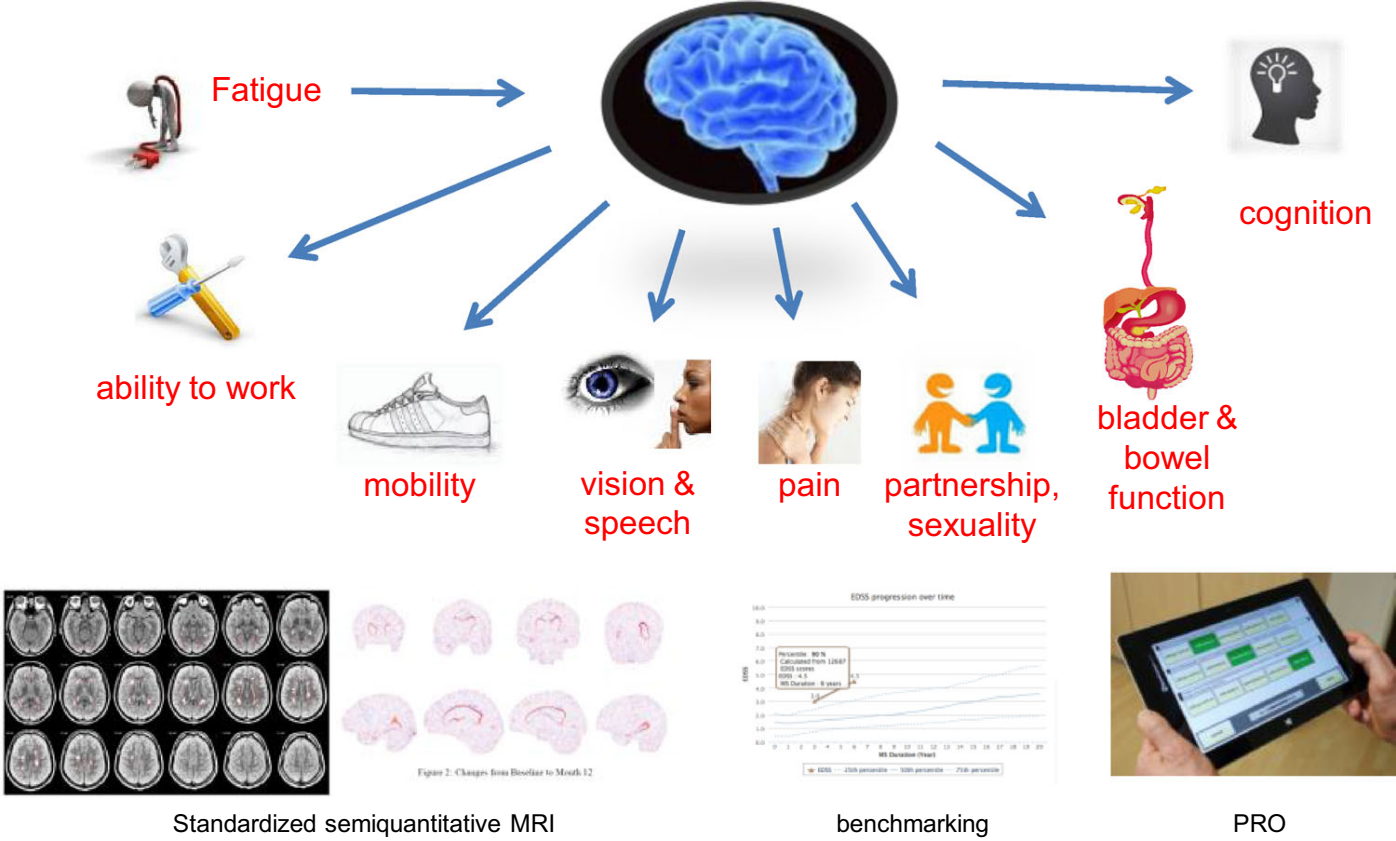
Assessment of patients at risk of disease progression or treatment failure will require attention to both traditional outcome measures, such as relapses and disability, and to newer measures such as MRI assessments of brain atrophy and patient-reported outcomes (PROs). This in turn will require benchmarking to establish baseline levels of disability, allowing longitudinal assessments of disability over time, and standardized MRI protocols to monitor treatment effects on brain atrophy

Based on the evidence currently available, a number of potential strategies for the management of MS can be defined, depending on the level of disease activity (Fig. 5). In patients with little evidence of disease activity at baseline, treatment can be started with conventional first-line therapies such as IFN-β, glatiramer acetate, DMF or teriflunomide. Treatment should be monitored at 6–12-month intervals, and highly effective agents such as fingolimod, natalizumab, or alemtuzumab substituted (subject to their licensing conditions) if signs of disease activity such as frequent relapses, increasing disability, or worsening MRI lesion burden (and possibly brain atrophy) are observed. For patients with highly active disease at baseline or rapidly evolving severe disease (≥2 disabling relapses in 1 year, with at least one Gd+ lesion on T1-weighted MRI or a significant increase in lesion load on T2-weighted MRI), newer agents can be used as first-line therapy, and treatment monitored to ensure that NEDA is achieved. While these strategies focus on clinical and MRI measures of disease activity, it should be noted that patient-reported progression of symptoms, adverse effects of treatment, and an inability to tolerate injections may also constitute grounds for switching treatments. There are currently no data to suggest that the early use of effective treatments presents a risk of ‘therapeutic burn-out.’ Rather, the clear evidence for a limited therapeutic window militates in favour of early intervention and treatment optimization. The increasing number of highly active treatments becoming available raises the possibility of sequential treatment where necessary.

## Conclusions

The introduction of highly effective treatments, such as fingolimod, natalizumab and alemtuzumab, has considerably expanded treatment options in MS. At the same time, the choice of treatment has assumed a new importance for a number of reasons. In particular, there is strong evidence that there is a limited time window to intervene effectively in patients with early MS, and that intervention during this period appears to be critical for achieving favourable long-term outcomes. Furthermore, a new therapeutic strategy, treating to target to achieve no evidence of disease activity, has emerged, and this may entail preservation of brain tissue in addition to the traditional endpoints of clinical relapses and MRI measures of inflammation. Importantly, treating to target necessitates regular monitoring of disease activity to allow prompt switches in cases of treatment failure.

Effective intervention during the window of opportunity requires identification of, and prompt response to, suboptimal response or treatment failure. However, it is difficult to define treatment failure adequately because much disease activity in MS (particularly during the early stages) is subclinical, and hence it is not usually possible to be sure that no disease activity is present and long-term consequences are—at least in part—unknown. It is therefore necessary to look for the best outcomes in groups of patients included in clinical trials in order to identify the most effective therapies. As emphasized above, it will also be necessary to monitor treatment with DMTs systematically and consistently in order to identify suboptimal response or treatment failure promptly. This will necessitate attention both to traditional clinical endpoints such as relapses and disability (with benchmarking of baseline levels of disability), and to newer outcome measures such as brain atrophy (measured using standardized MRI protocols, cognition and patient-reported outcomes (Fig. 5). The regular assessment of the patient can be supported by a computerized patient management system including PRO assessment, such as the MSDS 3D system [120], participation in registries which provide bench marking function, and standardized semi-quantitative MRI.

The evolving MS landscape, in which a number of new treatments are appearing—each with their own benefits and risks—will require a change in the nature of interactions between patients and their physicians, with a shared approach to clinical decision making that emphasizes patient-related goals. Together, these innovations in MS management offer exciting new opportunities to optimize treatment outcomes.
